# Treatment of Alkaline Cr(VI)-Contaminated Leachate with an Alkaliphilic Metal-Reducing Bacterium

**DOI:** 10.1128/AEM.00853-15

**Published:** 2015-07-21

**Authors:** Mathew P. Watts, Tatiana V. Khijniak, Christopher Boothman, Jonathan R. Lloyd

**Affiliations:** a School of Earth, Atmospheric and Environmental Sciences, Williamson Research Centre for Molecular Environmental Science, University of Manchester, Manchester, United Kingdom; b Institute of Microbiology, Russian Academy of Sciences, Moscow, Russia

## Abstract

Chromium in its toxic Cr(VI) valence state is a common contaminant particularly associated with alkaline environments. A well-publicized case of this occurred in Glasgow, United Kingdom, where poorly controlled disposal of a cementitious industrial by-product, chromite ore processing residue (COPR), has resulted in extensive contamination by Cr(VI)-contaminated alkaline leachates. In the search for viable bioremediation treatments for Cr(VI), a variety of bacteria that are capable of reduction of the toxic and highly soluble Cr(VI) to the relatively nontoxic and less mobile Cr(III) oxidation state, predominantly under circumneutral pH conditions, have been isolated. Recently, however, alkaliphilic bacteria that have the potential to reduce Cr(VI) under alkaline conditions have been identified. This study focuses on the application of a metal-reducing bacterium to the remediation of alkaline Cr(VI)-contaminated leachates from COPR. This bacterium, belonging to the Halomonas genus, was found to exhibit growth concomitant to Cr(VI) reduction under alkaline conditions (pH 10). Bacterial cells were able to rapidly remove high concentrations of aqueous Cr(VI) (2.5 mM) under anaerobic conditions, up to a starting pH of 11. Cr(VI) reduction rates were controlled by pH, with slower removal observed at pH 11, compared to pH 10, while no removal was observed at pH 12. The reduction of aqueous Cr(VI) resulted in the precipitation of Cr(III) biominerals, which were characterized using transmission electron microscopy and energy-dispersive X-ray analysis (TEM-EDX) and X-ray photoelectron spectroscopy (XPS). The effectiveness of this haloalkaliphilic bacterium for Cr(VI) reduction at high pH suggests potential for its use as an *in situ* treatment of COPR and other alkaline Cr(VI)-contaminated environments.

## INTRODUCTION

Chromium (Cr) is a significant component of contaminated soil and groundwater through a variety of environmental exposures from its widespread use in metallurgy and industrial processes (1–3). Under most environmental conditions, it is stable as the Cr(VI) and Cr(III) valence states (4). The Cr(III) state dominates under reducing conditions, forming largely insoluble Cr(III) hydroxide phases (5, 6), which are widely considered nontoxic (7). In contrast, the Cr(VI) species dominates under oxidizing conditions, forming the toxic, carcinogenic, and highly soluble oxyanions HCrO_4_^−^, CrO_4_^2−^, and Cr_2_O_4_^2−^ (8, 9). Due to the greater stability and mobility of Cr(VI) at high pH, it is particularly associated with contaminated alkaline environments (10). A well-known example of alkaline Cr(VI) contamination relates to the poorly controlled disposal of waste from the “high-lime” chromite ore (FeCr_2_O_4_) processing technique, chromite ore processing residue (COPR) (11, 12). Roasting the chromite ore with lime causes oxidation of Cr(III) to Cr(VI), enabling leaching with water (12). However, due to inefficiencies in the process, COPR contains significant concentrations of Cr, typically 3 to 7% by mass, of which 1 to 30% is typically in the Cr(VI) state (13, 14), while the addition of lime produces typically high pH values of 11 to 13 (15). The Cr(VI) forms part of a complex mineralogy, which upon saturation with groundwater readily yields alkaline leachate with high concentrations of aqueous Cr(VI) (15–17). COPR-related contamination is a global issue, with significant cases reported in the United Kingdom, United States, Eastern Europe, India, Pakistan, and China (11, 12, 18). For example, in Glasgow, United Kingdom, the poorly controlled disposal of >2 million metric tons of COPR has resulted in widespread contamination with highly alkaline Cr(VI) leachate, with groundwater and surface water containing up to 100 mg liter^−1^ (16, 19–21). These values are far in excess of the World Health Organization's upper limit for Cr(VI) in drinking water, 0.05 mg liter^−1^ (22).

A potential treatment of Cr(VI) contamination involves harnessing the microbial metabolism of bacteria that are capable of enzymatic metal reduction, reducing Cr(VI) to the relatively insoluble Cr(III) (23, 24). The ability to enzymatically reduce Cr(VI) has been observed among a diverse range of bacteria (25, 26), primarily among the facultative anaerobes (27). Microbial Cr(VI) reduction is often attributed to enzymes that have alternative metabolic functions (28), while a restricted range of bacteria are capable of using Cr(VI) as the terminal electron acceptor for growth (29, 30). Most previous studies have been carried out at near-neutral conditions, and pH extremes have proved a major limiting factor to enzymatic reduction (31). As Cr(VI) contamination is primarily associated with alkaline environments (4), several studies have sought to culture alkaliphilic bacteria capable of Cr(VI) reduction at high pH (32–37). Alkaliphiles exhibit optimum growth under alkaline conditions (pH 9 to 12) (38), while a number of these, the haloalkaliphiles, also require salinity for optimum growth (39). Haloalkaliphiles of the Halomonas genus are especially well represented in high-pH and high-salt environments (40, 41), and a number of studies have found these organisms to be capable of Cr(VI) reduction (34, 36). Halomonas species have also been reported for other remedial reactions, such as the reduction of nitrate (42), while the isolate used in this current study was also able to reduce the nuclear contaminant Tc(VII) to Tc(IV) (43).

Despite the identification of a small number of alkaliphilic bacteria capable of reducing Cr(VI) in model laboratory solutions, there remains a need to test these bacterial systems against environmental Cr(VI) contamination. The aim of this study is to determine whether the microbial metabolism of alkaliphilic bacteria can be harnessed for the reductive precipitation of Cr(VI) in high-pH leachates of COPR, modified with haloalkaliphilic medium and bacteria. This was explored by using a haloalkaliphilic soda lake isolate which has previously been reported to be effective at high-pH reduction of Tc(VII) (43). The findings of this study would therefore represent the first investigative results of the direct treatment of COPR leachates using an alkaliphilic Cr(VI)-reducing bacterium.

## MATERIALS AND METHODS

### Organism and culture conditions.

The facultative anaerobic haloalkaliphilic bacterium was originally isolated from Mono Lake (California, USA), herein referred to as the Mono Lake isolate, by N. N. Lyalikova (Institute of Microbiology, Russian Academy of Sciences, Moscow, Russia). The isolate was cultured using sterile anaerobic growth medium (pH 10) consisting of a basal medium of 13 g liter^−1^ Na_2_CO_3_, 4 g liter^−1^ NaHCO_3_, 50 g liter^−1^ NaCl, and 0.5 g liter^−1^ K_2_HPO_4_, and the following additional growth nutrients: 0.1 g liter^−1^ MgSO_4_·7H_2_O, 0.1 g liter^−1^ NH_4_Cl, 2 g liter^−1^ sodium acetate (Na acetate), 2 g liter^−1^ yeast extract, and 2 ml of mineral elixir (2.14 g liter^−1^ nitrilotriacetic acid, 0.1 g liter^−1^ MnCl_2_·4H_2_O, 0.3 g liter^−1^ FeSO_4_·7H_2_O, 0.17 g liter^−1^ CoCl_2_·6H_2_O, 0.2 g liter^−1^ ZnSO_4_·7H_2_O, 0.03 g liter^−1^ CuCl_2_·H_2_O, 0.005 g liter^−1^ AlKSO_4_·12H_2_O, 0.005 g liter^−1^ H_3_BO_3_, 0.09 g liter^−1^ Na_2_MoO_4_, 0.11 g liter^−1^ NiSO_4_·6H_2_O, and 0.02 g liter^−1^ Na_2_WO_4_·2H_2_O).

### COPR sample collection and preparation of a COPR extracted medium.

A sample of COPR was obtained from a borehole at a site in southeastern Glasgow, United Kingdom, transferred to a sterile plastic container, and stored in the dark for approximately 2 years at 10°C until use. The COPR has been extensively characterized in a previous study and found to be composed of a cementitous mineralogy, with considerable leachable Cr(VI) content (44, 45), consistent with previous studies (15, 17). To prepare the COPR extract medium, a subsample of field moist COPR was added to the basal medium at 5:100 (wt/vol) for growing cell experiments and 10:100 (wt/vol) for resting cell experiments, under aerobic conditions in the absence of any growth nutrients. The COPR medium slurry was homogenized by shaking and left in the dark at 20°C for 24 h to equilibrate. The resulting slurry was then filter sterilized using a 0.22-μm filter.

### Growing cell experiments.

Experiments using a growing culture of the Mono Lake isolate were conducted by inoculation of the previously stated 5:100 (wt/vol) COPR–basal medium extract in the presence of added growth nutrients. Growing cell cultures were prepared by mixing 11 ml of the filter-sterilized 5:100 COPR extract with 3.5 ml of sterile growth medium in sterile 20-ml serum bottles, which were then sealed using butyl rubber stoppers and aluminum crimps, in equilibrium with air. The serum bottles were then inoculated with a 1-ml aliquot of a growing anaerobic culture, maintained at 20°C, of the Mono Lake isolate. The bottles were then incubated at 30°C in the dark for the duration of the experiment. Samples (1 ml) were removed using a N_2_ degassed syringe and centrifuged (Sigma 1-14 Microfuge) at 13,000 × *g* for 5 min, and the supernatant was then removed for Cr(VI) analysis. The remaining solids were resuspended in 1% NaCl and again centrifuged (Sigma 1-14 Microfuge) at 13,000 × *g* for 5 min. The supernatant was then discarded and replaced with 100 μl of 1% NaCl, and the solution was homogenized for protein analysis.

### Resting cell experiments.

The isolate was also used in a series of anaerobic resting cell experiments in the presence of the 10:100 (wt/vol) COPR–basal medium extract as detailed in the medium preparation section. Under aerobic conditions, growth medium was inoculated with a culture of the Mono Lake bacterium and incubated in a sterile Erlenmeyer flask on a shaking incubator at 30°C. The culture was then harvested in late log phase (approximately 24 h) using a centrifuge (Sigma 6k15) at 5,000 × *g* for 20 min and washed three times using basal medium under an N_2_ atmosphere. The washed cells were then used to inoculate resting cell experiments to a final protein concentration of 81.5 μg ml^−1^ (equivalent to an optical density at 600 nm of 0.45). The resting cell experiments were composed of 20 ml of the COPR extract medium supplemented with 2 g liter^−1^ Na acetate and 2 g liter^−1^ yeast extract. All cultures were contained in sterile serum bottles sealed using butyl rubber stoppers and aluminum crimps and degassed using N_2_ gas passed through a 0.22-μm filter. Resting cell experiments were established at pH 10, 11, and 12 in triplicate, alongside noninoculated abiotic controls. The starting pH of the medium was 12, and the pH was subsequently adjusted to pH 11 and 10 in the corresponding cultures using sterile 3 M HCl. Aqueous samples were removed to monitor the geochemical parameters of the experiment using an N_2_ degassed syringe and centrifuged (Sigma 1-14 Microfuge) at 13,000 × *g* for 5 min, and a subsample of the supernatant was then analyzed for aqueous Cr(VI) concentration.

### Calculation of Cr(VI) removal reaction rates.

The Cr(VI) concentration data were fitted to a pseudo-first-order reaction rate model. The reaction rate is proportional to the concentration of aqueous Cr(VI), while the cell concentration is assumed to remain in far excess and constant throughout the experiment:

$$\frac{d\left[\text{Cr}\left(\text{VI}\right)\right]}{dt}=-k_{\text{obs}}\left[\text{Cr}\left(\text{VI}\right)\right]$$

where [Cr(VI)] is the concentration of aqueous Cr(VI), *t* is time, and *k*_obs_ is the observed first-order reaction rate constant. The first-order reaction rate model was applied only when significant aqueous Cr(VI) removal (>50%) was observed.

### Aqueous-phase analysis.

The pH of the samples was measured using a meter (Denver Instrument UB-10 meter) and a probe (Cole-Parmer 5990-45 CCP), calibrated using relevant pH buffers. The aqueous Cr(VI) concentration was determined by the 1,5-diphenylcarbazide (DPC) UV-visible light (UV-vis) spectrophotometric method and compared to K_2_CrO_4_ standards of known Cr(VI) concentration (46).

### Protein assay.

Protein concentrations were determined using a bicinchoninic acid (BCA) and Cu(II)SO_4_ spectrophotometric assay (47), quantified by comparison to bovine serum albumin (BSA) standards. All UV-vis measurements were recorded on a Jenway 6715 UV-vis spectrophotometer.

### Solid-phase analysis.

At the end of the resting cell experiment, the replicates that exhibited Cr(VI) removal were sampled for solid-phase analysis. The aqueous slurry was centrifuged (Sigma 1-14 Microfuge) at 13,000 × *g* for 5 min, the supernatant was removed and replaced with 18.2 MΩ water, and the resulting solution was homogenized. This was repeated three times, and the resulting pellet was dried in an anoxic glove box prior to analysis.

For transmission electron microscope (TEM) imaging, the pellet was resuspended in ethanol, and droplets were placed on an Agar Scientific holey carbon film grid and allowed to dry. The TEM analysis was performed on a Philips CM200 FEG TEM equipped with a field emission gun (FEG) and an energy-dispersive X-ray analyzer (EDX), Oxford Instruments X-Max 80-mm^2^ silicon drift detector (SDD) INCA EDX.

X-ray photoelectron spectroscopy (XPS) was performed on a Kratos Axis Ultra spectrophotometer with a monochromated Al Kα X-ray source. Analysis was carried out with an analyzer pass energy of 80 eV (wide scans) and 20 eV (narrow scans) with a total energy resolution of 1.2 and 0.6 eV, respectively, at a base pressure of 5 × 10^−8^ Pa. All spectra were fit with a Shirley background model (48) and had their photoelectron binding energies (BE) referenced to the C 1s adventitious carbon peak (285 eV BE). Fitting of the Cr 2p region was conducted using 70% Lorentzian and 30% Gaussian curves.

### DNA extraction, PCR amplification, and 16S rRNA gene sequencing.

The DNA of the isolate was extracted using a PowerSoil DNA isolation kit (MO BIO Laboratories, Inc., Carlsbad, CA, USA). The DNA was amplified by PCR using several broad-specificity 16S rRNA gene primers to obtain overlapping amplified 16S rRNA fragments, including 8F (49), 530F (50), 519R (51), 943R (50), and 1492R (51).

The dideoxynucleotide method was used to determine the nucleotide sequences (52), using an ABI Prism BigDye terminator cycle sequencing kit in combination with an ABI Prism 877 integrated thermal cycler and ABI Prism 377 DNA sequencer (PerkinElmer Applied Biosystems, Warrington, United Kingdom). A contig was generated from the sequences (typically 900 bp in length), using DNA Dragon v1.6 (SequentiX Digital DNA Processing, Klein Raden, Germany). The consensus sequence (1,446 bp in length) was analyzed against the NCBI (US) database using BLAST program packages and matched to known 16S rRNA gene sequences.

The phylogenetic tree was constructed using MEGA5 (53). The evolutionary history was inferred by using the neighbor-joining method (54), and the evolutionary distances were computed using the maximum composite likelihood method (55).

## RESULTS AND DISCUSSION

### Phylogenetic characterization.

**FIG 1:**
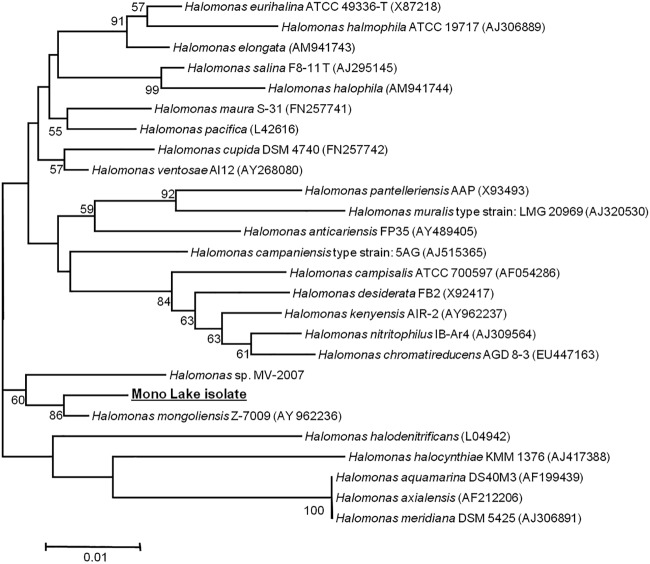
Phylogenetic tree based on 16S rRNA gene sequences of the Mono Lake isolate and other members of the Halomonas genus. GenBank nucleotide sequence accession numbers are in parentheses. The bar represents 1% divergence in the 16S rRNA gene sequence.

Sequencing of the 16S rRNA gene of the Mono Lake isolate showed that the organism belongs to the Halomonas genus of the Gammaproteobacteria (Fig. 1). The isolate occupies a clade with Halomonas mongoliensis and shares 16S rRNA gene sequence similarity to a variety of Halomonas species (Fig. 1).

Halomonas species are often highly represented in isolates from hypersaline and alkaline environments such as soda lakes (40, 41). These obligate heterotrophs have gained attention for other potentially useful behavior, such as their ability to degrade aromatic compounds (56–58) and produce alkaline enzymes with possible biotechnological applications (59, 60). In addition to this, a number of Halomonas species, including H. mongoliensis (61), the closest phylogenetic match to the Mono Lake isolate, have been found to be capable of anaerobic nitrate reduction under alkaline (pH 10) and high-salt conditions (4 M Na^+^) (36). Several closely related Halomonas strains have also been found to be capable of alkaline Cr(VI) reduction under anaerobic conditions (34, 36). As the Mono Lake isolate studied here has also been shown to reduce Tc(VII) to Tc(IV) (43), it may potentially reduce a variety of redox-active metals.

### Cr(VI) reduction during growth.

**FIG 2:**
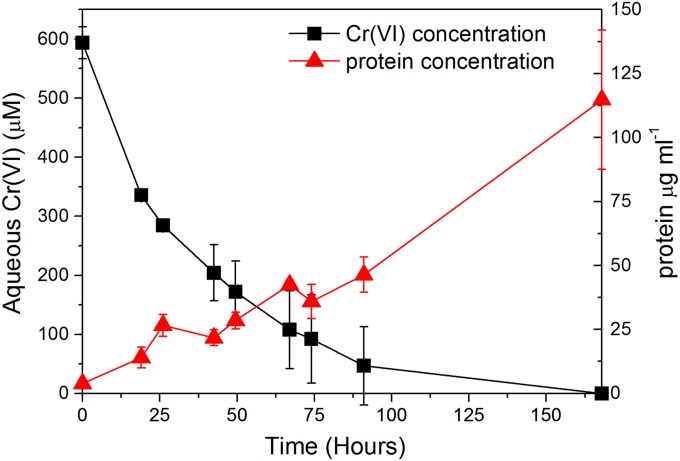
Aqueous Cr(VI) concentration and protein concentration after inoculation of the COPR leachate growth media with the Mono Lake Halomonas species. Error bars represent the standard deviations of duplicate experiments.

Upon inoculation of aerobic COPR extract on growth medium with the Mono Lake Halomonas species, the aqueous Cr(VI) concentration decreased to below detection limits within 170 h (Fig. 2), while the pH was maintained at 10 throughout the experiment. Concurrent to this removal of Cr(VI), protein concentrations increased steadily over the reaction period reaching more than 100 μg ml^−1^ after 168 h of incubation. This concurrent increase in protein levels, while Cr(VI) decreased, is clear evidence for growth of the isolate in the presence of significant Cr(VI) concentrations. As these cultures were initially incubated under oxic conditions, where the facultative anaerobic Halomonas species likely consumes O_2_ as an initial electron acceptor, it is unclear whether growth is directly coupled to Cr(VI) reduction.

### Cr(VI) reduction by resting cells.

**FIG 3:**
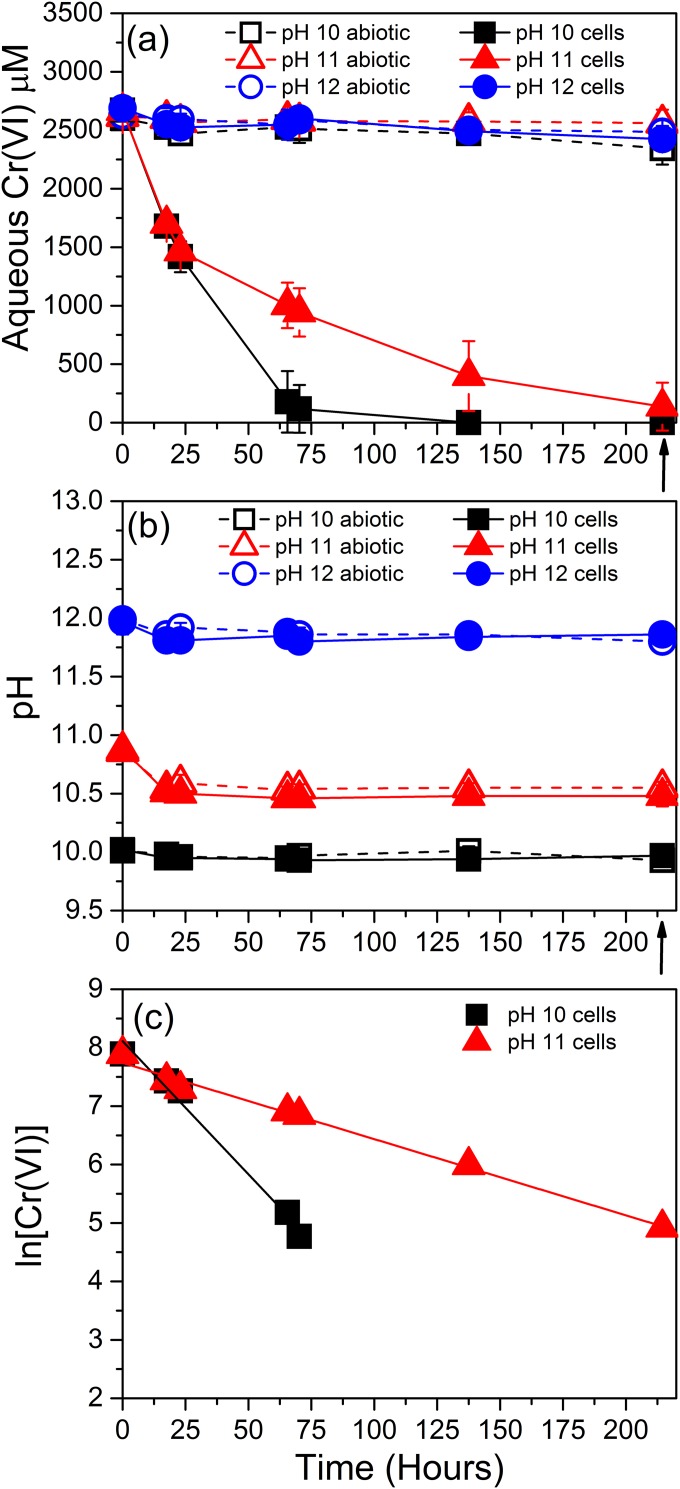
Aqueous Cr(VI) concentration (a) and pH (b) of the COPR leachate in sterilized controls (open symbols) and when inoculated with a late-log-phase culture of a Halomonas isolate from Mono Lake (solid symbols). The black arrows indicate sampling times for solid-phase analysis. Error bars represent the standard deviations of triplicates. (c) Pseudo-first-order Cr(VI) removal rate kinetics, plotted as ln [Cr(VI)] against time, calculated from the averages of triplicate Cr(VI) values of experiments containing resting cells of Halomonas sp. These experiments were performed at a starting pH of 10 and 11.

**FIG 4:**
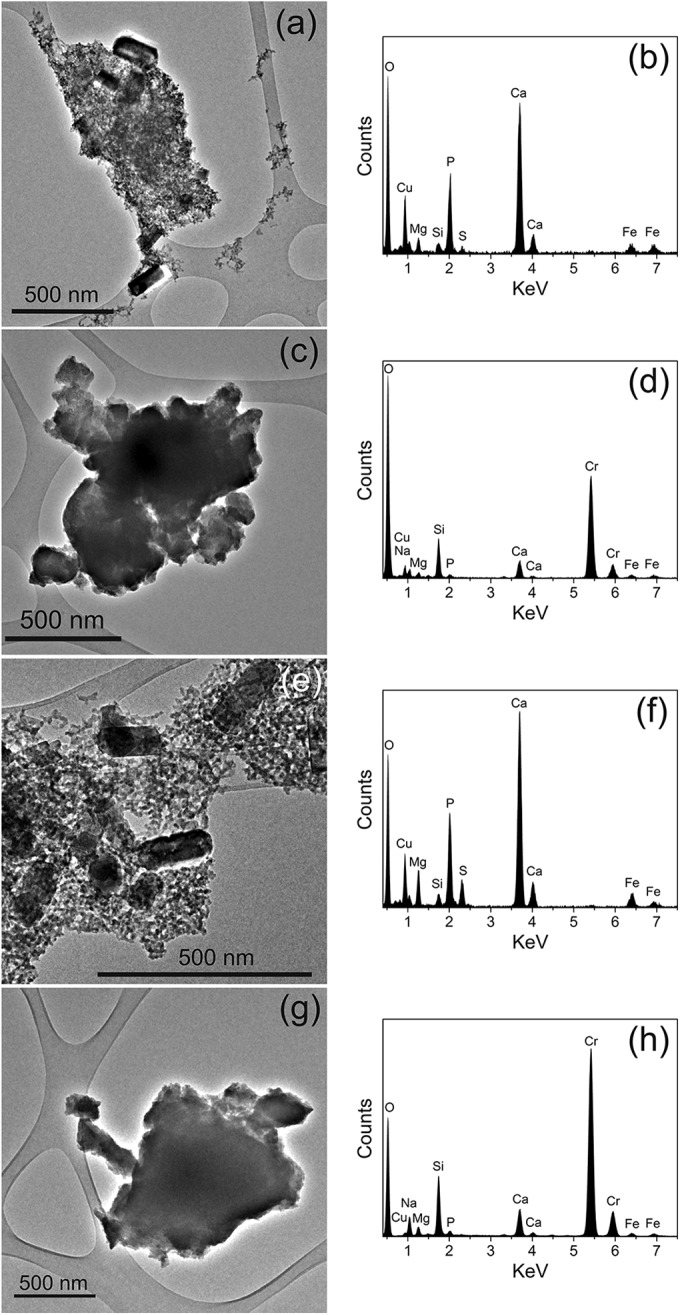
TEM images and their corresponding EDX spectra of Cr-containing solid phases from resting cell experiments at pH 10 (a to d) and pH 11 (e to h). The Cu component in the EDX spectra is due to its presence in the carbon-coated copper grids.

To identify the impact of pH on the kinetics of Cr(VI) reduction, a series of resting cell experiments were conducted at pH 10, 11, and 12 (Fig. 3). The Cr(VI) concentrations of cell-free abiotic control experiments showed little change over the duration of the experiment at all pH values, remaining at ∼2,500 μM throughout. However, rapid Cr(VI) removal was noted in the presence of cells of the Mono Lake Halomonas isolate in the cultures at pH 10 and 11, along with reductive precipitation of Cr(III), confirmed by XPS analysis of the resulting precipitates (Fig. 4 and Table 1). Thus, Cr(VI) removal was due to direct, anaerobic, enzymatic Cr(VI) reduction by the Mono Lake Halomonas isolate.

**TABLE 1:** Summary of XPS data obtained from precipitates formed by reduction of Cr(VI) by resting cell cultures of a Halomonas species from Mono Lake

Starting pH	XPS elemental composition (atomic %)								XPS valence state [Cr(III)/Cr(VI)]
	C	O	Cr	Na	Si	Ca	N	P	
10	67.8	20.3	0.4	0.4	5.4	0.2	6.0	0.3	100:0
11	73.0	21.7	0.6	0.2	3.3	0.5	0.8		74:26

The solution pH was found to have a strong control over enzymatic Cr(VI) reduction, with no appreciable Cr(VI) removal observed in the pH 12 replicate, suggesting a loss of metabolic activity under extremely alkaline conditions. The rapid removal of Cr(VI) noted at pH 10 and 11 translated to *k*_obs_ values that were considerably higher at pH 10 (0.0409 h^−1^; *R*^2^ = 0.96) compared to the pH 11 replicate (0.0126 h^−1^; *R*^2^ = 0.98) (Fig. 3c). As the starting pH of 11 evidently buffers down to 10.5 during incubation by the addition of the bacterial isolate, it is this level that should be assumed to be the upper limit of sustained Cr(VI) removal observed in this study. Reduction of Cr(VI) at this highly alkaline pH is in line with the upper limits of enzymatic Cr(VI) reduction reported previously (34, 36, 62, 63). These values are also consistent with previously reported optimum growth conditions of pH 9 to 10 for closely related Halomonas species (42, 61). The pH values of the COPR leachate and contaminated groundwater are typically within the range of 9 to 12.5 (16, 64). Therefore, the observed removal of Cr(VI) at alkaline values up to 10.5 indicates that a bioremediation approach using the Halomonas species may represent a possible treatment of a proportion of COPR leachates, without the need for pH amendment prior to inoculation, while treatment of higher pH COPR leachates would require some degree of buffering to a lower pH, albeit to a lesser extent than required for bioremediation using neutrophilic bacteria.

### Characterization of the Cr precipitates.

Upon visual inspection of the resting cell incubations at the end of the experiments, a purple precipitate was observed in the pH 10 cultures and a green precipitate was observed in the pH 11 cultures. The observed color differences in the precipitates would indicate the presence of differing precipitate phases where Cr(III) minerals can occur as green or purple minerals (65, 66). The fate of the Cr removed from solution in the resting cell experiments was assessed using TEM-EDX alongside XPS analysis.

**FIG 5:**
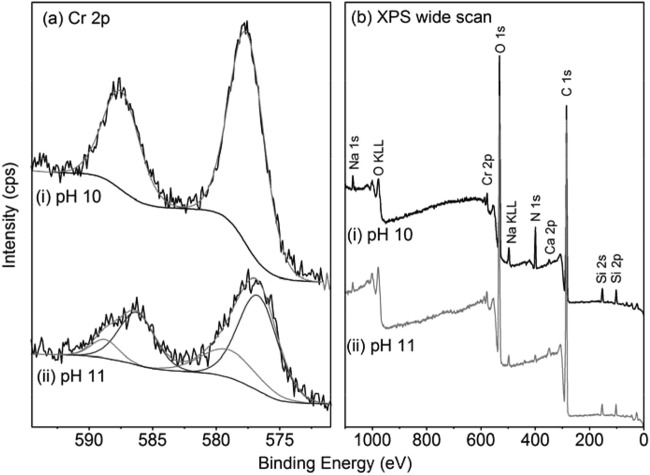
XPS Cr 2p region (a) and the wide-scan (b) spectra of the precipitates of the resting cell experiments at pH 10 (i) and 11 (ii).

The TEM images of the resulting precipitates and their corresponding EDX spectra are presented in Fig. 4, for the pH 10 (Fig. 4a to d) and pH 11 (Fig. 4e to h) incubations. The XPS wide-scan and Cr 2p region spectra are presented in Fig. 5, and their elemental composition and Cr valence states are presented in Table 1. TEM analysis shows that the precipitates formed at both pH 10 and pH 11 appear to possess a similar morphology, both containing a finer granular component (10- to 30-nm diameter) along with larger cubic structures (100 to 250 nm in length), visible in Fig. 4a and e. Both the precipitates formed at pH 10 and pH 11 also contained larger, micron-sized agglomerates (Fig. 4c and g), which appeared structurally featureless when viewed at high resolution. In addition to TEM-EDX analyses, the precipitates were probed using selected area electron diffraction (SEAD) (data not presented). However, the lack of ring structures noted indicated random diffraction commonly associated with amorphous structures.

The Cr 2p XPS region (Fig. 5a) was fitted with a component consisting of two peaks, at ∼576.5 and ∼586.0 eV BE, consistent with previously reported Cr(III) phases and a component of higher BE peaks, at ∼579.0 and ∼588.8 eV, consistent with Cr(VI) phases (67–69). The pH 10 precipitate was best fitted with the single Cr(III) component, while the pH 11 precipitates required fitting with a contribution from the Cr(VI) component, equating to 26% Cr(VI) of total Cr (Fig. 5 and Table 1). It is not clear whether the presence of Cr(VI) occurs as an adsorbed phase or within the bulk mineral, where due to the surface sensitivity of XPS, any adsorbed phase would be overrepresented in the spectra. The presence of Cr(VI) in the experiments exhibiting a lower rate of reduction is presumably due to incomplete reduction prior to sampling for solid-phase analysis.

The chemistry of the bulk sample was observed, by XPS, to be dominated by the C and O 1s regions, with smaller Si, Ca, Na, N, and Cr components. The relative contributions of these smaller components showed minimal variation between the two samples. It is important to note that the elemental composition noted by XPS reflects that of the bulk precipitates and not the Cr biominerals alone. Also, due to the surface sensitivity of XPS, typically sampling to a depth of <10 nm (70), the results are likely to overrepresent the finer-grained fraction as opposed to the bulk of larger Cr biominerals. The elemental chemistry of the samples was therefore analyzed using TEM-EDX to target the differing minerals present in the heterogeneous precipitates. The TEM-EDX spectra (Fig. 4b and f) do not show a significant Cr component and are composed primarily of Ca, P, and O, with minor contributions of S, Si, Fe, Na, and Mg. The larger micron-scale particles, from their corresponding EDX spectra (Fig. 4d and h), were principally composed of Cr and O, with minor contributions from Si and Ca. Relative intensities differed marginally between the pH replicates. The dominance of the Cr and O in the spectra would be consistent with the presence of a Cr(III) oxide or hydroxide, the latter being widely reported as the dominant form of Cr(III) in high-pH environments (1). However, the techniques employed in this study are not able to determine the exact phase of the Cr(III) and give little indication of possible differences indicated by the differing colors of the precipitates noted at pH 10 and 11.

### Conclusions.

This study has demonstrated the ability of an haloalkaliphilic soda lake isolate, belonging to the Halomonas genus, to grow while reducing aqueous Cr(VI) from a COPR extract under alkaline conditions. Bacterial cells were also able to anaerobically reduce significant concentrations of Cr(VI) (2.5 mM) under nongrowing conditions, with an inverse relationship between pH and reaction rates over the pH range tested here. Cr(VI) reduction occurred up to pH 10.5 and ultimately resulted in the precipitation of predominantly Cr(III) minerals.

The application of this Mono Lake Halomonas isolate offers a potential *in situ* bioremediation treatment for the reduction of alkaline leachates with high Cr(VI) concentrations associated with COPR contamination. These findings therefore provide useful information for the development of *in situ* trials via bioaugmentation of environments affected by COPR. In addition, as this organism has been demonstrated previously to have the ability to reduce Tc(VII) (43) and is closely related to other nitrate-reducing (61) and aromatic-degrading (58) strains of the Halomonas genus, this isolate may be of use in the bioremediation of a variety of contaminants occurring in alkaline environments.
